# Cloning and Sequence Analysis of LipL32, a Surface–Exposed Lipoprotein of Pathogenic Leptospira Spp

**DOI:** 10.5812/ircmj.8793

**Published:** 2013-11-05

**Authors:** Ebrahim Khodaverdi Darian, Mohammad Mahdi Forghanifard, Soheila Moradi Bidhendi, Yung-Fu Chang, Emad Yahaghi, Majid Esmaelizad, Maryam Khaleghizadeh, Pejvak Khaki

**Affiliations:** 1 Young Researchers and Elite Club, Karaj Branch, Islamic Azad University, Karaj, IR Iran; 2 Department of Biology, Damghan Branch, Islamic Azad University, Damghan, IR Iran; 3 Department of Microbiology, Razi Vaccine and Serum Research Institute, Karaj, IR Iran; 4 Department of Population, Medicine and Diagnostic sciences, College of Veterinary Medicine, Cornell University, Ithaca, USA; 5 Chemical Injuries Research Center, Baqiyatallah University of Medical Sciences, Tehran, IR Iran; 6 Department of Biotechnology, Razi Vaccin and Serum Resaerch Institute, Karaj, IR Iran

**Keywords:** Leptospirosis, Leptospira Interrogans, LipL32

## Abstract

**Background:**

Leptospirosis is a worldwide zoonosis caused by pathogenic Leptospira species. A major challenge of this disease is the application of basic research to improve diagnostic methods and related vaccine development. Outer membrane proteins of Leptospira are potential candidates that may be useful as diagnostic or immunogenic factors in treatment and analysis of the disease.

**Objectives:**

To develop an effective subunit vaccine against prevalent pathogenic Leptospira species, we sequenced and analyzed the LipL32 gene from three different Leptospira interrogans (L.interrogans) vaccinal serovars in Iran.

**Materials and Methods:**

Following DNA extraction from these three serovars, the related LipL32 genes were amplified and cloned in the pTZ57R/T vector. Recombinant clones were confirmed by colony- PCR and DNA sequencing. The related sequences were subjected to homology analysis by comparing them to sequences in the Genbank database.

**Results:**

The LipL32 sequences were >94% homologous among the vaccinal and other pathogenic Leptospira serovars in GenBank. This result indicates the conservation of this gene within the pathogenic Leptospires.

**Conclusions:**

The cloned gene in this study may provide a potentially suitable platform for development of a variety of applications such as serological diagnostic tests or recombinant vaccines against leptospirosis.

## 1. Background

Leptospirosis is recognized as the most widespread zoonosis with a global distribution, and is caused by Leptospira, a genus of spirochetal bacteria, spread either through direct contact of infected animals or through urine of hosts (1). Nearly 250 Leptospira serovars are classified into 24 serogroups (2) based on the expression of surface-exposed lipopolysaccharides (LPS) (3).Genetic categorization of Leptospira through DNA hybridization analyses indicates 13 pathogenic and six saprophytic species (1, 3-5). Seven of these species, including L.interrogans, L.borgpetersenii, L.santarosai, L.noguchii, L.weilli, L.kirschneri, and L.alexanderi, are the main causes of leptospirosis (6). Leptospirosis in humans is transmitted by either direct contact with infected animals or indirect contact with water, moist soil, or vegetation contaminated with urine from a host with chronic renal infection (7). The related clinical presentation in humans is difficult to distinguish from dengue, malaria, influenza, and many other diseases characterized by fever, headache, and myalgia (1, 8).

Protective immunity elicited by leptospiral lipopolysaccharide is generally serovar-specific (7). More than 250 different Leptospira serovars are known to exist and current available whole cell vaccines cannot protect against all of them (8, 9). Therefore, identification of a conserved molecule within all serovars that can be readily detected by the immune system may aid in the development of a subunit vaccine. Proteins expressed during infection may serve as determinants in leptospiral pathogenesis and as targets for the host immune response. The identification of leptospiral antigens expressed during infection has potentially important implications for the development of new serodiagnostic and immunoprotective strategies (7). In obligate intracellular bacteria, outer membrane proteins (OMPs) play crucial roles in the adaptation process by facilitating interactions between bacterial cells and their hosts (9). Therefore, identification and characterization of OMP components is not only essential in developing new subunit vaccines, but can also help to characterize suitable antigens for early diagnosis of the disease (10).

LipL32 is a major outer membrane lipoprotein that is highly conserved among pathogenic Leptospira species (10-12). Furthermore, this protein is the immunodominant antigen recognized during the humoral immune response to leptospirosis in humans (13).

## 2. Objectives

Molecular cloning techniques provide a tool for producing bulk quantities of antigens. With the long-term goal of developing an effective subunit vaccine against prevalent pathogenic Leptospira species, our aim in this study was to elucidate nucleotide variations and analyze the LipL32 gene sequences from three different L.interrogans vaccinal serovars in Iran, to make a pilot step towards the utilization of this gene for the production of recombinant antigen and its subsequent use in the diagnosis of leptospirosis.

## 3. Materials and Methods

### 3.1. Leptospira Isolates and Culture Conditions

Three vaccinal serovars of Linterrogans, including serovars Grippotyphosa (RTCC2808), Sejroe Hardjo (RTCC2821), Canicola (RTCC2805), and a saprophytic serovar biflexa (RTCC2819) were utilized in this study. All strains were obtained from the Leptospira Reference Laboratory of Microbiology Department, Razi Vaccine and Serum Research Institute, Karaj, Iran. The leptospiral serovars were cultivated separately in Ellinghausen-McCullough-Johnson-Harris (EMJH) semisolid medium (Difco, Sparks, USA) containing 1% bovine serum albumin, 2% rabbit serum, 0.1% sodium pyruvate, and 100 μg/mL 5-flurouracil in aerobic condition at 28°C. After 5-6 days, the isolates were harvested by centrifugation (Eppendorf, Germany) at 15000 × g for 20 minutes at 4°C.

### 3.2. Extraction of Genomic DNA

Genomic DNA was extracted by the standard phenol-chloroform method described by Sambrook and Russell (14). The quality of the extracted DNA was checked by ethidium bromide (EtBr) staining on agarose gel electrophoresis and analyzed spectrophotometrically using the Epoch system (BioTek, New York, USA).

### 3.3. PCR Amplification

To amplify the 835 bp LipL32 coding sequence we developed a specific primer pair (Table 1). 

**Table 1.** PCR Primer Sequences Used to Amplify LipL32 Genes Studied in This Work

Primer	Oligonucleotide Sequence
LipL32F ^a^	5´ CCTAACTAAGGAGAGTCTATG 3´
LipL32R^a^	5´ TTACTTAGTCGCGTCAGAAGC 3´

^a^ Abbreviations: F, forward primer; R, reverse primer

The PCR mixture contained 100 ng of template DNA, 10 pM of each primer, 200 μM mix of dNTPs, 1.5 mM MgSO_4_, 1xPCR buffer, and 0.25 U Pfu DNA polymerase (Fermentas, Lithuania) in a total volume of 50 μL PCR was performed under the following thermal profile: heat denaturation at 93°C for 4 minutes, 35 cycles of 93°C for 1 minute, 52°C for 1 minute, and 72°C for 1 minute, and the final extension at 72°C for 10 minutes, on a thermal cycler (Eppendorf, Germany). PCR products were electrophoresed on 1% agarose gels and stained with EtBr. Gels were photographed using a gel documentation system (Bio-Rad Chemi XRS Gel Documentation system, UAS). The specificity of the PCR was evaluated using DNA from non-pathogenic serovars as templates.

### 3.4. Gel purification and Cloning of PCR Products

The PCR products were purified from the gel using a DNA purification kit (Roche, Germany) and ligated into the pTZ57R/T vector (Fermentas, Lithuania) according to the manufacturer’s instructions. Briefly, the ligation reaction was incubated overnight at 4 °C following 10 minutes at 22 °C and the mixture was transformed into E.coli JM107 competent cells (obtained from the microbial culture collection of Leptospira Department of Microbiology, Razi Vaccine and Serum Research Institute, Karaj) using a chemical transformation protocol (14). The cells were then plated onto LB agar containing ampicillin, IPTG, and X-gal and incubated at 37 °C overnight. Following blue/white colony screening, recombinant colonies were confirmed by colony PCR and related plasmids were isolated from bacterial cells using the DNA plasmid Mini extraction kit (Roche, Germany) according to the manufacturer’s instructions.

### 3.5. Nucleotide Sequencing and Homology Analysis

The recombinant plasmids were sequenced. (Millegen Company, France). Nucleotide sequences have been deposited in the GenBank database of NCBI with the accession numbers JN831363, JN886739, and JN886738. The percentage of homology and divergence among examined leptospiral serovars was deduced using the MegAlign programme of Lasergene software DNASTAR. Based on the homology analysis, a phylogenetic tree was constructed using the Clustal method. Available sequences of L.interrogans serovars Sejroe Hardjo, Canicola, and Grippotyphosa were obtained from GenBank at the National Centre for Biotechnology Information (NCBI) website. Homology searches with the LipL32 sequences of different epidemic Leptospira serovars were accomplished using the BLAST program against sequences in the GenBank/NCBI nucleic acid sequence database.

## 4. Results

### 4.1. PCR Amplification and Cloning of LipL32 Gene

**Figure 1.**
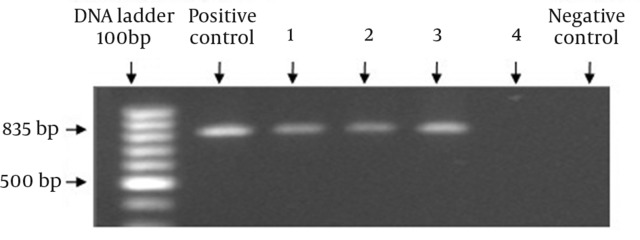
PCR amplification of the 835 bp LipL32 gene of L.interrogans serovars: 100 bp DNA ladder, Positive control: serovar Pomona, Lane1: serovar Grippotyphosa, Lane 2: serovar Sejroe Hardjo, Lane3: serovar Canicola, Lane 4: saprophytic serovar Biflexa, and negative control.

We amplified the LipL32 coding sequence from three different Leptospira serovars using a specific primer pair. The PCR amplified an 835 bp LipL32 product from all three pathogenic vaccinal serovars, but not from the non-pathogenic L.biflexa. This result confirmed the specificity of the designed primer pair to amplify LipL32 from pathogenic serovars (Figure 1). 

This result is consistent with previous studies (11, 13). Following gel purification, the PCR products were cloned into the pTZ57R/T vector and transformed into E.coli JM107 cells. Recombinants were confirmed by picking white colonies and carrying out colony PCR amplification of the LipL32 gene.

### 4.2. Homology Search and Sequence Analysis

**Figure 2.**
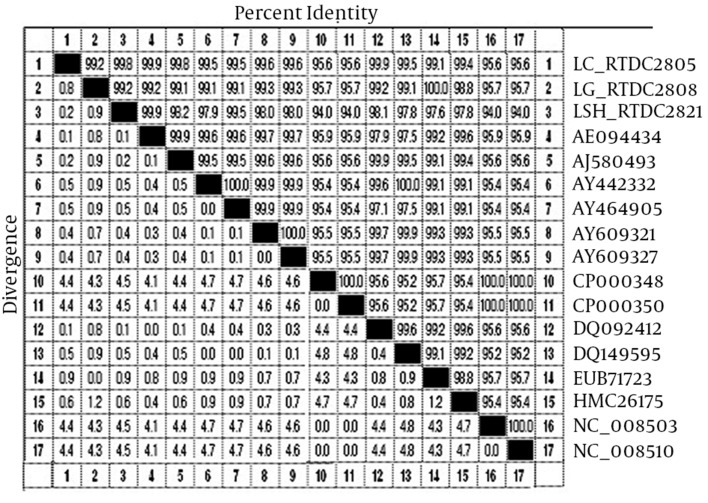
Sequence Pair Distances of LipL32 Gene Sequences of Different Leptospiral Serovars

**Figure 3.**
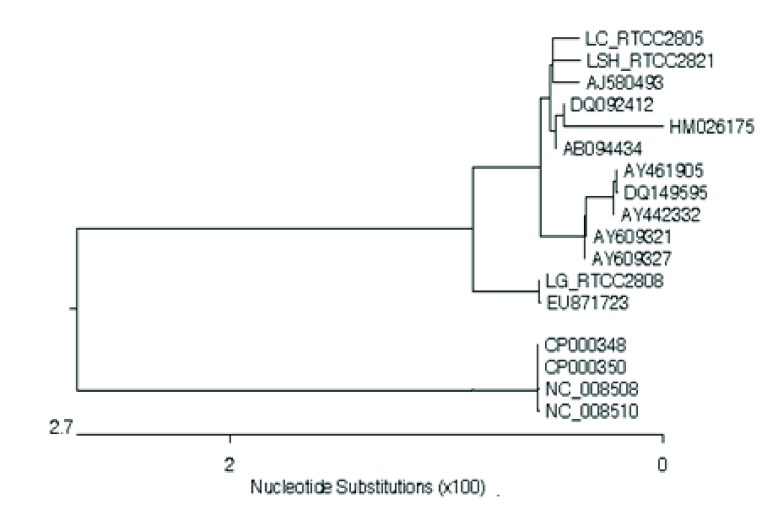
Phylogenetic Tree Analysis of LipL32 Gene

The LipL32 gene sequences of L.interrogans serovars Sejroe Hardjo, Canicola, and Grippotyphosa were obtained from GenBank. The features of the sequences are shown in Table 2. Homology searches with the LipL32 sequences of vaccinal serovars were accomplished using the BLAST program against the GenBank database. Homology analyses of the LipL32 sequences showed >94% similarity among the vaccinal leptospiral serovars (Figure 2). In this study, we found that sequences of the LipL32 gene in three examined vaccinal leptospiral serovars are identical with reference sequences previously submitted in GenBank (Figure 2 and 3) (15). Sequence analysis of the LipL32 gene of the vaccinal serovars revealed that serovar Sejroe (RTCC2821) is most closely related to Canicola (RTCC2805), while it is more distantly related to serovar Grippotyphosa (RTCC2808) with 99.8% and 99.2% homology, respectively (Figure 2 and 3). Moreover, this serovar is closely related to L.interrogans serovars Hardjo prajitno (AY461905, AY442332) and Sejroe (DQ149595) with > 98% homology. All three serovar (Hardjo prajitno (AY461905, AY442332) and Sejroe (DQ149595) serovars have been classified as belonging to a single genomospecies of L.interrogans.16 However, the results showed that L.borgpetersenii serovars Hardjo-bovis (NC_008510, NC_008508, CP000350, and CP000348) has minimal homology with our serovar Sejroe (RTCC2821) (Figure 3). Furthermore, L.interrogans serovar Canicola (RTCC2805) is most closely related to serovar Canicola (AB094434, DQ092412) isolated from Japan and India with 99.9% identity, while it is more distantly related (95.4% identity) to serovar Canicola (AY763509) isolated in China. The LipL32 sequence in Figure 2 shows that vaccinal serovar Grippotyphosa of L.interrogans (RTCC2808) is 100% identical to serovar Grippotyphosa in Malaysia (EU871723), forming a similar cluster. 

**Table 2.** BLAST Search Results of LipL32 Genes of Leptospira Spp Used for Phylogenetic Analysis

Organism	Serovar	Strain	Accession No.	Country
**L. interrogans**	Grippotyphosa	Lin 6	AY609327	China
**L. interrogans**	Grippotyphosa	Moskva V major	EU871723	Malaysia
**L. interrogans**	Canicola	Lin	AY609321	China
**L. interrogans**	Canicola	-	HM026175	India
**L. interrogans**	Canicola	-	DQ092412	India
**L. interrogans**	Canicola	Hond Utrecht IV	AJ580493	India
**L. interrogans**	Canicola	Hond Utrecht IV	AB094434	Japan
**L. interrogans**	Hardjo	Hardjoprajitno	AY461905	USA
**L. interrogans**	Hardjo	-	AY442332	India
**L. interrogans**	Sejroe	-	DQ149595	India
**L. borgpetersenii**	Hardjo-bovis	JB197	NC_008510	USA
**L. borgpetersenii**	Hardjo-bovis	L550	NC_008508	USA
**L. borgpetersenii**	Hardjo-bovis	L550	CP000348	Australia
**L. borgpetersenii**	Hardjo-bovis	JB197	CP000350	USA

## 5. Discussion

Leptospirosis, caused by different Leptospira species, is one of the most widespread zoonotic infections worldwide (1, 15). A major challenge in combating this disease is the application of basic research to improve diagnostics and vaccine development. Diagnosis is complicated because, on one hand, the available diagnostic tests are not always serovar-specific due to cross-reactivity against different serovars that may occur between organisms in the same serogroup. On the other hand, protein expression of pathogenic leptospires differs when grown outside vs. inside the host. For these and other reasons, the molecular mechanisms of the pathogenesis of leptospirosis remain unclear. Several candidate virulence factors have been identified that may contribute to the pathogenesis of Leptospira infections. These include LPS, outer membrane proteins (OMPs) and other surface proteins, and adhesion molecules. Among these, OMPs may be potential targets to induce and enhance immune responses against the disease. One of the most important OMPs expressed during human infection is LipL32, a 32-kDa lipoprotein that is highly conserved in pathogenic species but absent in non-pathogens (11). LipL32 binds to different extracellular matrix proteins such as collagens I, IV, and V, as well as laminin (11, 16). Furthermore, LipL32 demonstrated a calcium-dependent fibronectin binding activity (16, 17). Therefore, due to LipL32’s important roles in pathogenesis, its conserved sequence among different pathogenic species may present an ideal target for vaccine production, especially considering that there are currently no widely available vaccines against leptospirosis for use in humans. Nonetheless, virulence factors such as LigA and LigB are being considered as possible vaccine targets. In this study, having considered OMPs as important immunogenic components to develop subunit vaccines against the Leptospira pathogens, we analyzed the LipL32 sequences from different domestic pathogenic Leptospira species and compared them to related sequences from other species. LipL32 from the examined species had not been sequenced previously in Iran and our study is the first report focusing on domestic prevalent pathogenic Leptospira. Our results showed that the LipL32 sequence of L.interrogans is highly similar to that of L.borgpetersenii. This result agrees with other studies that found the LipL32 sequence of the L.interrogans strain was identical to the related sequence L.borgpetersenii strains. Our results also indicate that LipL32 is strictly conserved among the pathogenic Leptospires, in accordance with previous studies (10, 11, 18, 19). In humans, resistance to leptospirosis is largely achieved through humoral immunity in which the main target of produced antibodies is leptospiral LPS (1, 2, 20). However, immunity induced by anti-LPS antibodies is relatively serovars-specific, and therefore efficient only against disease caused by homologous, or antigenically similar, serovars. Unlike anti-LPS antibodies, whole leptospiral protein preparations are also used to induce immunity against Leptospira species. Such preparations illustrated remarkable protection against challenge with either homologous or heterologous Leptospira serovars, although side effects are inevitable (2). In addition, combinational use of leptospiral OMPs such as OmpL1 and LipL41 were introduced as protective immunogens (21), which is promising in the development of protective subunit vaccines with few side-effects. Here we analyzed the LipL32 sequence from different pathogenic Leptospira and showed its high similarity among the species. These results may shed light on the use of this gene to produce a subunit vaccine with widely protective immunity. In addition, the presented data and the results may serve an acceptable platform to produce recombinant LipL32 protein, which can further be used as an antigen either in serologically ELISA-based tests or in the production of related specific antibody using invivo systems. Because of similarities in symptoms, leptospirosis is often misdiagnosed as influenza, aseptic meningitis, encephalitis, dengue fever, hepatitis, or gastroenteritis. However, there are ways to detect and inhibit the progress of infection. The most important issue is timely diagnosis, because prompt and specific treatment early as early as possible in the illness is critical to ensure a favorable clinical outcome. Various serological tests are available to detect the infection. The standard reference test for the detection of leptospiral-specific antibodies is the microscopic agglutination test (MAT). Although the MAT is specific, it is also laborious and uses live leptospires as antigens, causing problems in antigen standardization and posing potential danger to laboratory personnel (22). Recombinant protein-based ELISA is a suitable and safe serological procedure for the examination of a large number of sera as it involves an immunodominant antigen and lacks nonspecific moieties present in whole-cell preparations (23). Because the LipL32 protein is highly conserved among the pathogenic serovars of Leptospira (11). It can serve not only as a specific antigen with high sensitivity for different serological tests, but it also offers promise as a possible immunogenic antigen to develop a subunit vaccine against Leptospira infections. Here we analyzed the LipL32 coding sequence from three L.interrogans vaccinal serovars (Grippotyphosa, Sejroe Hardjo, Canicola). In agreement with other reports that described similar findings (10, 11, 24), our results showed more than 94% similarity within the three pathogenic Leptospira strains. Therefore the LipL32 protein could be a useful diagnostic tool for detection of leptospirosis in animals. In conclusion, this study showed high similarity of the LipL32 gene sequences between our vaccinal serovars and reference sequences in GenBank. This indicates that the LipL32 gene is highly conserved among different pathogenic leptospiral serovars. The cloned gene in this study offers a potentially suitable platform for the development of a variety of applications, such as serological diagnostic tests or recombinant vaccines against leptospirosis.
